# Prevalence and factors associated with inadequate work ability among community health workers in Montes Claros, Minas Gerais state, Brazil: a cross-sectional study, 2018

**DOI:** 10.1590/S2237-96222024v33e2023354.en

**Published:** 2024-04-05

**Authors:** Jamile Pereira Dias dos Anjos, Ronilson Ferreira Freitas, Karine Suene Mendes Almeida, Antônio Prates Caldeira, Daniela Araújo Veloso Popoff, Josiane Santos Brant Rocha

**Affiliations:** 1 Universidade Estadual de Montes Claros, Programa de Pós-Graduação em Cuidado Primário em Saúde, Montes Claros, MG, Brazil; 2 Universidade Federal do Amazonas, Faculdade de Medicina, Manaus, AM, Brazil; 3 Universidade Estadual de Montes Claros, Departamento de Enfermagem, Montes Claros, MG, Brazil

**Keywords:** Work ability Assessment, Community Health Worker, Occupational Health Surveillance, Health Personnel, Cross-sectional Studies, Evaluación de la Capacidad de Trabajo, Agente de Salud Comunitario, Vigilancia de la Salud del Trabajador, Personal de Salud, Estudios Transversales, Avaliação da Capacidade de Trabalho, Agente Comunitário de Saúde, Vigilância em Saúde do Trabalhador, Pessoal de Saúde, Estudos Transversais

## Abstract

**Objective:**

To estimate the prevalence and analyze factors associated with inadequate work ability among community health workers (CHWs).

**Methods:**

This was a cross-sectional study conducted with CHWs, from July to October 2018, in Montes Claros, state of Minas Gerais, Brazil; work ability, sociodemographic, occupational, and clinical factors were investigated; prevalence ratios (PRs) with 95% confidence intervals (95%CI) were calculated using Poisson regression.

**Results:**

Of the 675 CHWs, 25.8% (95%CI 22.7;29.2) showed inadequate work ability; length of service greater than five years (PR = 1.64; 95%CI 1.24;2.18), poor health status (PR = 2.10; 95%CI 1.56;2.83), depressive symptoms (PR = 1.98; 95%CI 1.54;2.55) and voice disorders (PR = 1.85; 95%CI 1.26;2.73) were associated with the event.

**Conclusion:**

There was a high prevalence of inadequate work ability, associated with occupational and clinical factors.

## Study contributions

**Main results:**

There was a high prevalence of inadequate work ability among community health workers (CHWs), associated with occupational and clinical factors.

**Implications for services:**

This study can contribute to the planning of preventive actions and the promotion of the work ability of CHWs, with repercussions on the quality of service provided by these professionals.

**Perspectives:**

Longitudinal studies are strongly recommended in order to establish cause-and-effect relationships between the variables investigated.

## RESUMO

**Objetivo:**

Estimar a prevalência e analisar fatores associados à capacidade inadequada para o trabalho entre agentes comunitários de saúde (ACS).

**Métodos:**

Estudo transversal realizado com ACS, no período de julho a outubro de 2018, em Montes Claros, Minas Gerais, Brasil; foi investigada a capacidade para o trabalho, fatores sociodemográficos, laborais e clínicos; razões de prevalência (RPs) com intervalos de confiança de 95% (IC_95_%) foram calculadas por regressão de Poisson.

**Resultados:**

Dos 675 ACS estudados, 25,8% (IC_95_% 22,7;29,2) apresentaram capacidade inadequada para o trabalho; tempo de serviço superior a cinco anos (RP = 1,64; IC_95_% 1,24;2,18), percepção do estado de saúde ruim (RP = 2,10; IC_95_% 1,56;2,83), sintomas depressivos (RP = 1,98; IC_95_% 1,54;2,55) e distúrbios da voz (RP = 1,85; IC_95_% 1,26;2,73) estiveram associados ao evento.

**Conclusão:**

Houve prevalência elevada de capacidade inadequada para o trabalho, associada a fatores laborais e clínicos.

## RESUMEN

**Objetivo:**

Estimar la prevalencia y analizar factores asociados a la inadecuada capacidad para el trabajo entre los agentes comunitarios de salud (ACSs).

**Métodos:**

Estudio transversal realizado con ACS, de julio a octubre de 2018, en Montes Claros, Minas Gerais, Brasil; se investigó la capacidad de trabajo y factores sociodemográficos, ocupacionales y clínicos; las razones de prevalencia (RP) con intervalos de confianza del 95% (IC_95_%) se calcularon mediante regresión de Poisson.

**Resultados:**

De los 675 ACS, el 25,8 % (IC_95_% 22,7;29,2) presentó capacidad laboral inadecuada; tiempo de servicio superior a 5 años (RP = 1,64; IC_95_% 1,24;2,18), mal estado de salud (RP = 2,10; IC_95_% 1,56;2,83), síntomas depresivos (RP = 1,98; IC_95_% 1,54;2,55) y trastornos de la voz (RP = 1,85; IC_95_% 1,26;2,73) estaban relacionados con el evento.

**Conclusión:**

Hubo alta prevalencia de capacidad inadecuada para el trabajo, asociada a factores laborales y clínicos.

## INTRODUCTION

In recent decades, workers’ health has been a matter of concern regarding changes in their profile and the economically active population ageing, which has repercussions on work ability.^1,2^ This is a result of a dynamic process of interaction between human resources and work, which can change over time,^2,3^ and is an indicator of workers’ health.^4,5^

Work ability refers to a worker’s competence or ability to perform their job, taking into account the demands required, their health status, and their physical and mental health conditions.^1,2,5^ There is a close and complex relationship between health and work, and health is negatively affected when job duties are performed in environments that are inappropriate and capable of precipitating or aggravating a reduction in work ability.^1^

Several factors are associated with work ability, including sociodemographic characteristics,^2,6^ health status,^7^ behavioral,^6,8,9^ and clinical characteristics,^7,6,10^ work conditions and organization.^6,8,9^

Work ability has been investigated in some categories, especially nurses and nursing technicians working in the hospital settings,^1,11^ as well as teachers.^4^ However, there are few studies on work ability in the context of Family Health Strategy (FHS) teams, particularly community health workers (CHWs). The essential role of CHWs in providing healthcare to users of the Brazilian National Health System (*Sistema Único de Saúde* - SUS) calls for further studies on this group and their relationship with work; CHWs often face inadequate working conditions and overwhelming workloads.^12^ Evidence shows that if healthcare professionals have adequate work ability, the healthcare provided to the population in primary health care will be better.^6,10,11^ Assessing the working conditions of CHWs can contribute to the planning of preventive actions and the promotion of work ability, in addition to generating interest among other researchers in this topic.

The objective of this study was to estimate the prevalence and analyze factors associated with inadequate work ability among CHWs.

## METHODS

This was a cross-sectional study conducted with CHWs in the municipality of Montes Claros, seat of the Northern health macro-region of Minas Gerais state, Brazil. This region represents a transition area between the most developed region in the country, the Southeast region, and the least developed region, the Northeast region, characterized by a vast territory of economic disparities.

CHWs who were registered with an FHS team and performed their duties in the municipality of Montes Claros in 2018 were invited to take part in the study. Those who were on leave, reassigned to other duties or on sick leave were excluded. Sample size calculation was performed to determine the size of the population of participating CHWs, using a 95% confidence level and a margin of error of 5% as parameters. Given the investigation of several outcomes, an expected prevalence of the phenomenon equal to 50% was used to obtain the largest sample size. The statistical power of the contingent of participating CHWs (type ꞵ error) was performed using *post-hoc* test, for comparison purpose among the groups related to the variables analyzed.

Data collection took place at the Regional Occupational Health Reference Center (*Centro de Referência Regional em Saúde do Trabalhador* - CEREST), from August to October 2018, conducted by a specially trained team, comprised of nurses, physicians, nutritionists, physical education professionals and undergraduate students. The participants answered a self-administered, printed questionnaire constructed based on published studies^5,13^ addressing sociodemographic, occupational and clinical information.

The outcome variable “work ability” was assessed using an instrument, the Work Ability Index (WAI), which has already been validated for Brazilian Portuguese. The WAI comprises seven dimensions: (1) Current work ability compared with lifetime best (one question); (2) Physical and mental job demands (two questions); (3) Current diseases diagnosed by a physician (one question/list of 56 diseases); (4) Estimated work impairment due to diseases (one question); (5) Sick leave during the past year (one question); (6) Own prognosis about work ability two years from now (one question); and (7) Mental resources (three questions).^5^ The WAI score was calculated by adding up the points for each dimension, and the final score ranged from 7 (the worst index) to 49 (the best index), categorized as follows: low (7 to 27); moderate (28 to 36); good (37 to 43); and excellent (44 to 49). Subsequently, the WAI was dichotomized: inadequate work ability (low and moderate indices); Adequate work ability (good and excellent indices).^5^

Sedentary behavior was measured using data from the International Physical Activity Questionnaire (IPAQ)^14^ related to the sum of time spent sitting, considering both weekdays and weekends. A weighted average was calculated as follows: the time on weekdays multiplied by 5, added to the time on weekends multiplied by 7, to obtain the average number of hours per day spent in a sitting position.^15^ In this context, the variable was dichotomized based on the average time spent sitting: up to 4 hours; more than 4 hours.

Self-perceived health was assessed by asking the following question, extracted from the Vigitel survey instrument:^16^ *Compared to people your age, how do you consider your health status?.*

There were four answer options: very good; good; regular; poor. For analysis purposes, the variable “self-perceived health status” was dichotomized: “good” (answer options: very good; good) or “poor” (answer options: regular; poor).

Body mass index (BMI) was calculated using measurements of height and weight obtained during data collection. Height was measured using a SECA 206® stadiometer, with a precision of 0.1 cm and a maximum capacity of 2.05 m, fixed to a wall at a 90-degree angle to the floor and without baseboards. The CHWs were asked to stand in an orthostatic position, with their gaze directed forward. For weight measurement in kilograms (kg) a BALMAK 111® mechanical anthropometric medical scale, with a precision of 100 g and a capacity of 300 kg was used, and the CHWs were wearing light clothing. BMI was calculated by dividing body weight by height squared (W/H^2^).^17^ As no underweight individuals were identified for analysis purposes, participants had their BMI categorized as normal (BMI < 25 kg/m²) or overweight/obesity (≥ 25 kg/m²).

Self-esteem was assessed using the Rosenberg Self-Esteem Scale (RSES), adapted and validated by Hutz^18^ for the Brazilian population. The score was obtained by means of a Likert scale, offering the following response options: strongly agree (0 point); agree (1 point); disagree (2 points); strongly disagree (3 points). The final self-esteem score ranged from 0 (worst situation) to 30 points (best situation), and was dichotomized into two levels: high self-esteem (< 15); low self-esteem (≥ 15).

Depressive symptoms were assessed using the Patient Health Questionnaire-9 (PHQ-9),^19^ an instrument that evaluates depression symptoms occurring in the 14 days prior to the assessment. For each PHQ-9 question, there are four response options: “not at all” (0 point); “several days” (1 point); “more than half the days” (2 points); “nearly every day” (3 points). The final score is classified into five groups:^24^ 0 to 4 points (no symptoms); 5 to 9 points (mild depression); 10 to 14 points (moderate); 15 to 19 points (moderately severe); and 20 to 27 (severe).^19^ In this study, a final score greater than 9 was considered as the cut-off point for the presence of depression symptoms.^19^

Voice disorder was assessed using the Screening Index for Voice Disorder (SIVD), designed to screen individuals with possible voice disorders. This instrument is comprised of 12 items: hoarseness, loss of voice, breaking voice, low-pitched voice, throat clearing, dry cough, cough with phlegm, pain when speaking, pain when swallowing, secretion in the throat, dry throat and fatigue when speaking. Validated for the Brazilian population of teachers, although it has been applied to other professional categories, including CHWs,^20^ the SIVD is comprised of a Likert scale, with scores up to 4, based on the following response options: never; rarely; sometimes; always.^20^ For analytical purposes, this variable was dichotomized: present or absent, and voice disorder was considered present when the response was “sometimes” and “always”.^20^

The presence of arterial hypertension was assessed based on participants’ self-report.

The duration of sun exposure was obtained from the following question: *How long do you spend in the sun during the day?,* with the following response options in hours: 1 to < 2; 2 to < 4; 4 to < 5; and > 5.^21^ Subsequently, the response was dichotomized: 1 to 4 hours; more than 4 hours.

Finally, the study variables were grouped into three blocks:

a) Sociodemographic
  - sex (male; female);
  - race/skin color (Black; White);
  - marital status (with a partner; without a partner);
  - schooling (higher education; elementary education);
  - and family income [in minimum wage (minimum wage in 2018 = BRL 954.00), categorized as follows: less than 1 minimum wage; 1 minimum wage or more].
b) Occupational
  - work ability [according to the work ability index: inadequate (low/moderate WAI);
  - adequate (good/excellent WAI)];
  - training in the healthcare field (yes; no);
  - length of time working as a CHW (in years: up to 5; more than 5); and
c) Clinical
  - sedentary behavior (hours/day spent in a sitting position: up to 4; more than 4);
  - high blood pressure (yes; no);
  - self-perceived health status (good; poor);
  - body mass index (BMI) (normal; overweight/obesity);
  - self-esteem (high; low);
  - depressive symptoms (yes; no);
  - time spent in the sun (hours/day: 1 to 4; more than 4);
  - and presence of voice disorders (yes; no).

Data were analyzed using the Statistical Package for the Social Sciences (SPSS), version 21.0. Initially, a descriptive analysis of the frequency distribution (absolute and relative) of work ability according to participants’ characteristics was performed. Subsequently, a bivariate analysis of the association between the factors studied and inadequate work ability was carried out by calculating the crude prevalence ratio (PR) and its respective 95% confidence interval (95%CI), along with Pearson’s chi-square test. Variables that showed an association with the outcome at a significance level of ≤ 20% were included in multiple Poisson regression models with robust variance. The criterion for remaining in the final model was associations with a significance level of 5% (p-value < 0.05). The variables in the final model were selected using the backward stepwise strategy.

In compliance with the National Health Council (CNS), Resolution No. 466, of October 12, 2012, the study was submitted to the Research Ethics Committee of the Universidade Estadual de Montes Claros (CEP/Unimontes) and was approved: Opinion No. 2,425,756. Before starting data collection, participants read and signed the Free and Informed Consent Form.

## RESULTS

A total of 797 CHWs working in primary health care in the municipality were invited to take part in the study; of these, 122 (15.3%) were excluded due to reassignment to other duties, being pregnant and/or on pregnancy leave, working in the position for less than one year, or being on sick leave, resulting in a final sample of 675 CHWs. No refusals were identified, and the comparative analysis of the main characteristics of the participating group in relation to the excluded group did not show any statistically significant differences. The post-hoc power calculation was found to be above 80%.

Table 1 shows the descriptive analysis of the sociodemographic, occupational, and clinical characteristics of the population surveyed. Among the 675 CHWs, the average age was 36.71 ± 9.85, with a predominance of females, Black race/skin color, being without a partner, education level up to elementary school, and family income higher than 1 minimum wage. Regarding inadequate work ability, a prevalence of 25.8% (95%CI 22.7;29.2) was observed.

**Table 1:** Variables related to work ability, sociodemographic, occupational and clinical characteristics of community health workers (n = 675), Montes Claros, state of Minas Gerais, Brazil, 2018

Characteristic variables		Total		Inadequate work ability	
		**N**	**%**	**n**	**%**
**Sociodemografic**					
Sex					
	Male	110	16.3	14	12.7
	Female	565	83.7	160	28.3
Race/skin color					
	Black	588	87.1	148	25.2
	White	87	12.9	26	29.9
Marital status					
	With a partner	403	59.7	118	29.3
	Without a partner	272	40.3	56	20.6
Schooling					
	Higher education	292	43.3	64	21.9
	Elementary education	383	56.7	110	28.7
Family income (in minimum wages)^a^					
	> 1	629	93.2	168	26.7
	≤ 1	46	6.8	06	13.0
**Occupational**					
Trainning in the healthcare field					
	Yes	241	35.7	83	34.4
	No	434	64.3	91	21.0
Length of time working as a CHW (in years)					
	≤ 5	382	56.6	58	15.2
	> 5	293	43.4	116	39.6
**Clinical**					
Sedentary behavior (hours spent sitting)					
	≤ 4	390	57.8	108	27.7
	> 4	285	42.2	66	23.2
Arterial hypertension					
	Do not have	604	89.5	146	24.2
	Have	71	10.5	28	39.4
Self-perceived health status					
	Good	398	59.0	55	13.8
	Poor	277	41.0	119	43.0
Body mass index – BMI					
	Normal	264	39.1	58	22.0
	Overweight/obesity	411	60.9	116	28.2
Self-esteem					
	High self-esteem	408	60.4	103	25.2
	Low self-esteem	267	39.6	71	26.6
Depressive symptoms					
	Eperience	547	81.0	98	17.9
	Do not experience	128	19.0	76	59.4
Time spent in the sun (in hours)					
	1-4	238	35.3	47	19.7
	> 4	437	64.7	127	29.1
Presence of voice disorders					
	No	200	29.6	24	12.0
	Yes	475	70.4	150	31.6

a) Minimum wage: BRL 954.00 in 2018.

Table 2 presents the results of the crude and adjusted analyses of the association between work ability and the independent variables considered. In the bivariate analysis, sex, marital status, schooling, family income, training in the healthcare field, length of time working as a CHW, self-reported arterial hypertension, self-perceived health status, presence of depressive symptoms, sun exposure time, and presence of voice disorders were statistically associated with inadequate work ability. In the adjusted analysis, the following remained associated with inadequate work ability: working as a CHW for more than five years (PR = 1.64; 95%CI 1.24;2.18), poor self-perceived health status (PR = 2.10; 95%CI 1.56;2.83), experiencing depressive symptoms (PR = 1.98; 95%CI 1.54;2.55) and presenting voice disorders (PR = 1.85; 95%CI 1.26;2.73).

**Table 2:** Crude and adjusted analysis for sociodemographic, occupational and clinical factors and inadequate work ability among community health workers (n = 675), Montes Claros, state of Minas Gerais, Brazil, 2018

Factors		Crude PR^a^ (_95_%CI^b^)	p-value^c^	Adjusted PR^a^ (_95_%CI^b^)	p-value^d^
**Sociodemographic**					
Sex					
	Male	1.00	0.002	1.00	0.079
	Female	2.22 (1.34;3.69)		1.55 (0.94;2.54)	
Race/skin color					
	Black	1.00	0.337	–	**–**
	White	1.18 (0.83;1.68)		–	
Marital status					
	With a partner	1.00	0.013	–	**–**
	Without a partner	0.70 (0.53;0.92)		–	
Schooling					
	Higher education	1.00	0.048	–	**–**
	Elementary education	1.31 (1.00;1.71)		–	
Family income (in minimum wages)^d^					
	> 1	1.00	0.064	–	**–**
	≤ 1	0.48 (0.22;1.04)		–	
**Occupational**					
Trainning in the healthcare field					
	Yes	1.00	< 0.001	–	**–**
	No	0.60 (0.47;0.78)		–	
Length of time working as a CHW (in years)					
	≤ 5	1.00	< 0.001	1,00	0.001
	> 5	2.60 (1.97;3.43)		1.64 (1.24;2.18)	
**Clinical**					
Sedentary behavior (hours spent sitting)					
	≤ 4	1.00	0.187	–	**–**
	> 4	0.83 (0.64;1.09)		–	
Arterial hypertension					
	Do not have	1.00	0.003	–	**–**
	Have	1.63 (1.18;2.24)		–	
Self-perceived health status					
	Good	1.00	< 0.001	1.00	< 0.001
	Bad	3.10 (2.34;4.11)		2.10 (1.56;2.83)	
Body mass index (BMI)					
	Normal	1.00	0.074	–	**–**
	Overweight/obesity	1.28 (0.97;1.69)		–	
Self-esteem					
	High self-esteem	1.00	0.695	**–**	**–**
	Low self-esteem	1.05 (0.81;1.36)		–	
Depressive symptoms					
	Do not experience	1.00	< 0.001	1.00	< 0.001
	Experience	3.31 (2.63;4.16)		1.98 (1.54;2.55)	
Time spent in the sun (in hours)					
	1-4	1.00	0.010	–	**–**
	> 4	1.47 (1.09;1.97)		–	
Presence of voice disorders					
	No	1.00	< 0.001	1.00	0.002
	Yes	2.73 (1.82;4.10)		1.85 (1.26;2.73)	

a) PR: Prevalence ratio; b) 95%CI: 95% confidence interval; c) p-value: Probability of significance - Pearson’s chi-square test; d) p-value = probability of significance - final model of multiple analysis, adjusted for the variables “length of service”, “self-perceived health status”, “depressive symptoms” and “voice disorders”; d) Minimum wage: BRL 954.00 in 2018.

## DISCUSSION

Among the CHWs working in the FHS in Montes Claros, state of Minas Gerais, more than a quarter showed inadequate work ability. Having been in this position for more than five years, having a poor self-perceived health status, experiencing depressive symptoms and presenting voice disorders were associated with this outcome.

These results are relevant, taking into consideration that they are a significant portion of CHWs in Montes Claros. Higher prevalence of inadequate work ability was found in studies conducted with CHWs in the municipality of Uberaba, also in Minas Gerais state,^3^ and in João Pessoa, the capital city of the state of Paraíba;^10^ In both studies, more than half of the CHWs studied showed impaired work ability.^3,10^ A study carried out among CHWs in Juiz de Fora, Minas Gerais state,^6^ and another in Pato Branco, state of Paraná,^22^ the latter conducted with workers in the furniture industry, also showed high prevalence levels of the WAI.^6,22^ In the context of primary health care, CHWs stand out as one of the professional categories responsible for preventive and health promotion actions, strengthening the bond between the population and services.^6,10,12^ Therefore, the high prevalence of inadequate work ability found in this study may lead to significant damage to the care provided to the population.

The study sample, predominantly female, confirms the prevalence of this sex in roles related to healthcare assistance and caregiving, in addition to reaffirming that working as a caregiver is a role culturally associated with women.^10^ It is worth considering the hypothesis that this question about the gender distribution among the CHWs reflects the community’s potential resistance to male professionals in this position. Living in the same area where they work, male CHWs might face challenges in certain situations, such as discussing intimate female and/or family issues during home visits, which could potentially cause embarrassment for residents and/or their families.^10^ Although being female was not associated with inadequate work ability, the predominance of female professionals in the study population emphasizes the need for greater attention to CHWs, given that women’s responsibilities extend beyond the workplace to include the demands of their homes.

Regarding the clinical characteristics of the CHWs, the majority of participants did not show sedentary behavior, despite the high prevalence of overweight/obesity observed, which is in line with other studies.^23,24^ According to Barbosa et al.,^10^ the prevalence of obesity contributes to the onset of other morbidities and impaired work ability; however, this association was not identified in this study.

Multiple regression analysis showed that length of service exceeding five years, self-perceived health status, presence of depressive symptoms and voice disorders were associated with the outcome in the final model.

With regard to the length of time working as CHWs, as reported in a systematic literature review, there is a trend towards worsening work ability as the length of time this professional spent in the role extends throughout life.^1^ This result can be explained by the fact that more time spent working implies more exposure to work-related risks, which can trigger diseases and other disorders that affect work ability.^5^ However, the effect of length of service in reducing the work ability of CHWs is not uniformly accepted among researchers. Some authors argue that longer working time promotes greater engagement with the profession,^24^ enhances the establishment of bonds and users’ trust in the work of CHWs, contributing to the best performance of their duties.^6,10,12^

Poor self-perceived health status was associated with a higher prevalence of inadequate work ability, corroborating studies conducted with different professional categories.^4,7^ As discussed in an assessment of the work ability among civil servants at a higher education institution in Southern Brazil,^7^ health was one of the factors that most positively influenced higher productivity at work. Self-rated health status is a crucial predictor of early retirement, as it evaluates the individual in their physical, mental, and social aspects. The influence of health status on work ability is evident, and it is considered a fundamental resource for professionals to reach their productive potential.^4^

Data from this study showed that the prevalence of inadequate work ability was approximately twice as high among CHWs who experienced depressive symptoms, when compared to those without these symptoms. This finding is similar to that of another study with CHWs, conducted in the municipality of Juiz de Fora, Minas Gerais state.^6^ In another study with CHWs, conducted in the municipality of Uberaba, also in Minas Gerais state,^3^ lower scores assessing the impact of emotional problems on work and mental health of individuals were significantly associated with reduced work ability.

According to Martinez et al.,^11^ the way work is organized can positively or negatively influence occupational health, emphasizing the need to establish a balance between expectations and reality at work to promote the mental health of professionals. Mental disorders in CHWs may result from the nature and overload of their duties, as well as the conditions in which they are performed,^25^ potentially leading to impairment, reduced effectiveness in their roles, and increased likelihood of taking sick leave.^6^

Depressive symptoms represent a serious public health problem, being a significant cause of disability and work absenteeism worldwide. The high prevalence of depressive symptoms among healthcare workers can have severe consequences, both for workers (absence from work, decreased productivity, suicide) and for service users (incompetence and omission to care).^25^ Therefore, mental health and work organization should be given priority in discussion and implementation of public policies, aiming at promoting the health of CHWs.

There was high prevalence of voice disorders among the CHWs studied, which were associated with inadequate work ability. A study conducted with CHWs in the east zone of the city of São Paulo^26^ identified high prevalence of complaints such as hoarseness, dry throat, shortness of breath and fatigue when speaking. These symptoms may be associated with the high vocal demand required in the duties performed by the CHWs, such as home visits, family registration and community meetings, making these professionals more susceptible to voice disorders. Inadequate environmental conditions for carrying out their duties – high ambient noise, temperature changes, presence of smoke and dust – in addition to the frequent need to speak loudly, when associated with low fluid intake, may contribute to these disorders.^22,26^ In addition, living in the same community where they work, CHWs can provide guidance after work hours, which intensifies the use of their voices.^26^

As for the limitations of this study, its cross-sectional design exposes it to the so-called “healthy worker effect”: workers on leave – on vacation or on sick leave – were excluded from the sample, which may have underestimated the work ability index. A second limitation is that, although cross-sectional studies enable the identification of risk or protective factors for work ability, they do not allow the establishment of cause-and-effect relationships. However, it is worth noting that the sample was representative of the CHW population and the variables investigated were obtained from previously validated questionnaires.

It can be concluded that the prevalence of inadequate work ability among CHWs was high, and that occupational and clinical factors were associated with this outcome. This assessment of the working conditions of CHWs can contribute to the planning of preventive actions and the promotion of work ability, as well as stimulate the interest of other researchers in the topic. Effective public health policies for the care of CHWs should be encouraged and implemented in order to prevent inadequate work ability of health workers in communities.

Finally, there is a need for public investment by the three levels of management in the SUS, aiming to provide better working conditions and value CHWs in line with the importance of their role in the context of primary health care for Brazilians.

## References

[1] Godinho MR, Ferreira AP, Fayer VA, Bonfatti RJ, Greco RM (2017). Capacidade para o trabalho e fatores associados em profissionais no Brasil. Rev Bras Med Trab.

[2] Rodrigues DDM, Aquino RL, Antunes DE, Costa MM, Oliveira PC, Aragão AS (2019). Work ability assessment for nursing team working at a large hospital in the region of triângulo mineiro – MG. Rev Min Enferm.

[3] Paula ÍR, Marcacine PR, Castro SS, Walsh IAP (2015). Work ability, musculoskeletal symptoms and quality of life among community health workers in Uberaba, Minas Gerais, Brazil. Saude Soc.

[4] Alcantara MA, Medeiros AM, Claro RM, Vieira MT (2019). Cad Saude Publica.

[5] Teixeira JRB, Mussi FC, Araujo TM, Boery EM, Casotti CA, Pereira R (2019). Factors associated with the work capacity of motorcycle taxi drivers. Cien Saude Colet.

[6] Moura DCA, Leite ICG, Greco RM (2020). Prevalência de sintomas de depressão em agentes comunitários de saúde. Trab Educ Saude.

[7] Amorim JSC, Mesas AE, Trelha CS (2018). Fatores associados à ótima capacidade para o trabalho em servidores idosos de uma universidade no Sul do Brasil. Rev Bras Saude Ocup.

[8] Gracino ME, Torjada JS, Castro-Alves MB (2018). Analysis of physicians work ability, in the city of Maringá, Brazil. Rev Bras Med Trab.

[9] Barreto CR, Lins-Kusterer L, Carvalho FM (2019). Work ability of military police officers. Rev Saude Publica.

[10] Barbosa AM, Lacerda DAL, Viana FDA (2019). Análise da capacidade para o trabalho de agentes comunitários de saúde em João Pessoa-PB. Rev Bras Cien Saude.

[11] Martinez MC, Latorre MRDO, Fischer FM (2017). Stressors influence work ability in different age groups of nursing professionals: 2-year follow-up. Cien Saude Colet.

[12] Costa EM, Ferreira DLA (2011). Percepções e motivações de agentes comunitários de saúde sobre o processo de trabalho em Teresina, Piauí. Trab Educ Saude.

[13] Alcantara MA, Duarte ACM, Simões MRL, Barroso HH, Barbosa REC, Fonseca GC (2023). Fatores associados a multicomorbidades autorreferidas em trabalhadores da rede de saúde municipal. Rev Bras Saude Ocup.

[14] Matsudo S, Araújo T, Matsudo VKR, Andrade D, Andrade E, Oliveira LC (2001). Questionário Internacional de Atividade Física (IPAQ): estudo de validade e reprodutibilidade no Brasil. Rev Bras Ativ Fis Saude.

[15] Rocha BMC, Godbaum M, César CLG, Stopa SR (2019). Sedentary behavior in the city of São Paulo, Brazil: ISA-Capital 2015. Rev Bras Epidemiol.

[16] Ministério da Saúde (BR) (2016). Vigitel Brasil 2016: vigilância de fatores de risco e proteção para doenças crônicas por inquérito telefônico.

[17] World Health Organization. (2000). Obesity: preventing and managing the global epidemic. Report of a World Health Organization Consultation.

[18] Hutz ﻿CS﻿﻿ (2000). ﻿Adaptação brasileira da Escala de Autoestima de Rosenberg.

[19] Santos IS, Tavares BF, Munhoz TN, Almeida LSP, Silva NTB, Silva NTB (2013). Sensibilidade e especificidade do Patient Health Questionnaire-9 (PHQ-9) entre adultos da população geral. Cad Saude Public.

[20] Murta JAN, Barbosa MS, Caldeira AP, Barbosa-Medeiros MR, Rossi-Barbosa LAR (2021). Factors associated with voice complaints in community health agents. CoDAS.

[21] Araújo FC, Sousa BRM, Leite GG, Freitas LC, Lemos ELC, Pires CAA (2016). Avaliação dermatológica de agentes comunitários de saúde sujeitos à fotoexposição em região tropical do Brasil. Sci Med.

[22] Linhares JE, Marcis J, Tonello R, Pessa SLR, Oliveira GA (2019). Evaluation of the Work Ability of workers in the furniture sector of a city in the south of Brazil. Gest Prod.

[23] Barbosa AM, Lacerda DAL (2017). Associação entre consumo alimentar e estado nutricional em agentes comunitários de saúde. Rev Bras Cien Saude.

[24] Ferraz L, Aerts DRGC (2005). O cotidiano de trabalho do agente comunitário de saúde no PSF em Porto Alegre. Cien Saude Colet.

[25] Silva ATC, Lops CS, Susser E, Menezes PR (2016). Work-related depression in primary care teams in Brazil. Am J Public Health.

[26] Cipriano FG, Ferreira LP (2011). Queixas de voz em agentes comunitários de saúde: correlação entre problemas gerais de saúde, hábitos de vida e aspectos vocais. Rev Soc Bras Fonoaudio.

